# CRISPRi for specific inhibition of miRNA clusters and miRNAs with high sequence homology

**DOI:** 10.1038/s41598-022-10336-3

**Published:** 2022-04-15

**Authors:** Monika Drobna-Śledzińska, Natalia Maćkowska-Maślak, Roman Jaksik, Paulina Dąbek, Michał Witt, Małgorzata Dawidowska

**Affiliations:** 1 Institute of Human Genetics, Polish Academy of Sciences, Strzeszynska 32, 60-479 Poznań, Poland; 2 Silesian University of Technology, Akademicka 16, 44-100 Gliwice, Poland

**Keywords:** Gene expression analysis, Genetic techniques, Non-coding RNAs, Gene expression, Gene regulation

## Abstract

miRNAs form a class of noncoding RNAs, involved in post-transcriptional regulation of gene expression, broadly studied for their involvement in physiological and pathological context. Inhibition of mature miRNA transcripts, commonly used in miRNA loss-of-function experiments, may not be specific in case of miRNAs with high sequence homology, e.g. miRNAs from the same seed family. Phenotypic effects of miRNA repression might be biased by the repression of highly similar miRNAs. Another challenge is simultaneous inhibition of multiple miRNAs encoded within policistronic clusters, potentially co-regulating common biological processes. To elucidate roles of miRNA clusters and miRNAs with high sequence homology, it is of key importance to selectively repress only the miRNAs of interest. Targeting miRNAs on genomic level with CRISPR/dCas9-based methods is an attractive alternative to blocking mature miRNAs. Yet, so far no clear guidelines on the design of CRISPR inhibition (CRISPRi) experiments, specifically for miRNA repression, have been proposed. To address this need, here we propose a strategy for effective inhibition of miRNAs and miRNA clusters using CRISPRi. We provide clues on how to approach the challenges in using CRISPR/dCas in miRNA studies, which include prediction of miRNA transcription start sites (TSSs) and the design of single guide RNAs (sgRNAs). The strategy implements three TSS prediction online tools, dedicated specifically for miRNAs: miRStart, FANTOM 5 miRNA atlas, DIANA-miRGen, and CRISPOR tool for sgRNAs design; it includes testing and selection of optimal sgRNAs. We demonstrate that compared to siRNA/shRNA-based miRNA silencing, CRISPRi improves the repression specificity for miRNAs with highly similar sequence and contribute to higher uniformity of the effects of silencing the whole miRNA clusters. This strategy may be adapted for CRISPR-mediated activation (CRISPRa) of miRNA expression.

## Introduction

miRNAs form a class of short noncoding RNAs, implicated in virtually all biological processes, by their involvement in post-transcriptional negative regulation of gene expression. They interact with their target mRNAs via complementary binding of their 6–8 nucleotide long seed sequence with miRNA response element (MRE) present primarily in 3′UTR of mRNA^1,2^. Yet, a growing body of evidence points to the importance of the nucleotide context of miRNA sequence beyond the seed pairing^3,4^. miRNAs belonging to the same seed family (having identical seed sequence) might recognize a different spectrum of target mRNAs^3,4^ and even small sequence differences can modulate target gene recognition^5^. To elucidate the potentially different roles of miRNAs with high sequence homology, it is of great importance to selectively repress only the miRNA of interest, without off-target repression of highly similar miRNAs.

miRNAs may be encoded as clusters in common genomic loci. Such miRNAs arise from one primary miRNA (pri-miRNA) transcript and may cooperate in the regulation of cellular pathways and processes. Thus, the repression of individual miRNAs may have modest effect on phenotype of the cells, while downregulation of the whole cluster of potentially cooperating miRNAs can result in much more profound effects^6^.

In miRNA loss-of-function experiments, a commonly used approach is the inhibition of a mature miRNAs. This might be achieved either temporarily (by small interfering siRNA/short hairpin shRNA inhibitors or miRNA sponges introduced to the cells via transfection)^7–9^ or constantly (shRNA or miRNA sponges encoding sequences introduced to the genome via transduction)^10–13^. These techniques may not be fully specific in case of miRNAs with highly homologous sequence and the observed phenotypic effects of miRNA repression may be biased by off-target repression of highly similar miRNAs. The transcript-based simultaneous inhibition of all miRNAs encoded within one cluster might also pose a challenge in terms of uniform effectiveness towards all miRNAs^14^.

One of the possible solutions to address these issues is the use of CRISPR-Cas9 technique to precisely target the miRNA coding region at the genome level. Cas9 nuclease is directed to the target site in the genome via complementary binding of a single guide RNA (sgRNA) molecule and introduce double stranded DNA breaks^15,16^. This approach has been widely used to knockdown the expression of miRNAs^17^, e.g. for disruption of Drosha-processing site and thus miRNA maturation^18,19^ or to study the interactions between miRNAs and their target genes by disruption of MRE^20^. DNA cleavage by Cas9 nucleases in off-target regions might lead to unexpected phenotypic effects^21,22^. Yet, DNA cleavage is not necessary—a broad spectrum of applications is available by using dCas9 (dead Cas9 protein—with switched off nuclease activity) particularly when fused with a desired effector domain, as reviewed by Anton et al.^23^.

In CRISPR interference approach (CRISPRi), dCas9 is directed by sgRNA to a promoter or a transcription start site (TSS) of a target gene and suppresses transcription by the sole fact of the occupation of this region and inhibiting the binding of transcription factors. dCas9 protein may be additionally fused with an effector domain (e.g. Krüppel associated box—KRAB) to strengthen the inhibitory effect. Although this technique has been extensively used for silencing of protein coding genes, there are only 6 reports on using CRISPRi in miRNA studies published over the last 8 years (first report published in 2014)^24–29^ in contrast to hundreds of published studies utilizing siRNAs/shRNAs.

The relatively infrequent use of CRISPRi and CRISPRa for the modulation of miRNA expression is mainly caused by the lack of clear guidelines on the CRISPRi/CRISPRa experimental design, adjusted specifically to miRNAs. One of the aspects that needs to be addressed is the knowledge on the location of miRNAs’ TSSs in the genome, which, in contrast to protein coding regions, is still insufficient. The precise location of miRNA promoter regions and TSS has only been annotated for a fraction of miRNAs, since the classical features of promoter regions may not be valid for miRNA promoter prediction^30^. The recognition of miRNA TSS via sequencing of pri-miRNA transcripts is particularly challenging, considering short half-life and rapid processing of these transcripts in the cells^31^. In recent years, databases collecting information about pri-miRNA, miRNA promoter and TSS location have been developed. Yet, the annotations for miRNA TSSs may differ depending on the database or algorithm used for its prediction.

**Figure 1:**
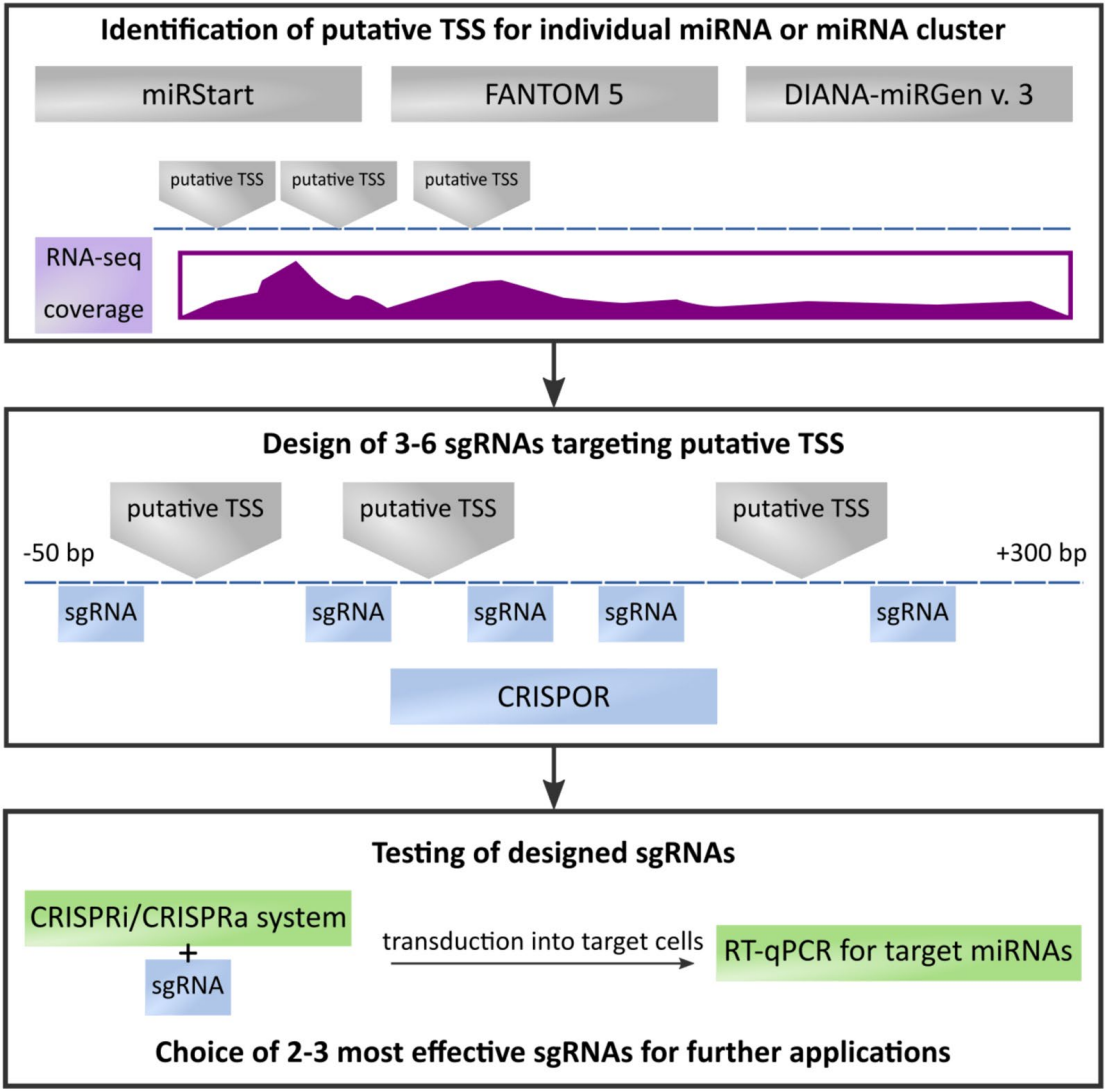
The strategy of the application of CRISPRi/CRISPRa approach for specific repression or activation of individual miRNAs or miRNA clusters. TSS—transcription start site.

Here we propose a strategy for an effective inhibition of miRNAs and whole miRNA clusters using CRISPRi. We provide clues on how to approach the biggest challenges in using CRISPR/dCas in miRNA studies, which are the prediction of miRNA TSS and the design of sgRNAs. The strategy we propose is depicted in Fig. 1. We show that this approach improves the repression specificity in case of mature miRNAs with highly similar but not identical sequence as compared to siRNA/shRNA-based miRNA silencing. We also postulate that the dCas9-mediated inhibition of miRNAs may be beneficial in case of studying whole miRNA clusters: targeting the TSS for the common pri-miRNA (on genomic level) may contribute to highly uniform effects towards all miRNAs within a cluster, as compared to simultaneous inhibition of individual clustered miRNAs (on mature transcript level). Our strategy may also be adapted for CRISPR-mediated activation (CRISPRa) of miRNA expression.

## Results

### Design of sgRNAs targeting TSS of individual miRNAs and miRNA clusters

To test the utility of CRISPRi for simultaneous inhibition of multiple clustered miRNAs, we selected 2 miRNA clusters: a prototypic oncogenic mir-17–92 cluster (located on chromosome 13) and a paralogous miRNA cluster mir-106a-363 (located on chromosome X). In Supplementary Table S1 we show miRNAs from these clusters and the third paralogous cluster mir-106b-25, highlighting their sequence homology. To test the specificity of CRISPRi towards highly similar miRNAs, we selected hsa-miR-130a-3p (with locus on chromosome 11) and hsa-miR-130b-3p (with locus on chromosome 22), which mature sequences differ by only two nucleotides (Supplementary Table S1).

**Figure 2:**
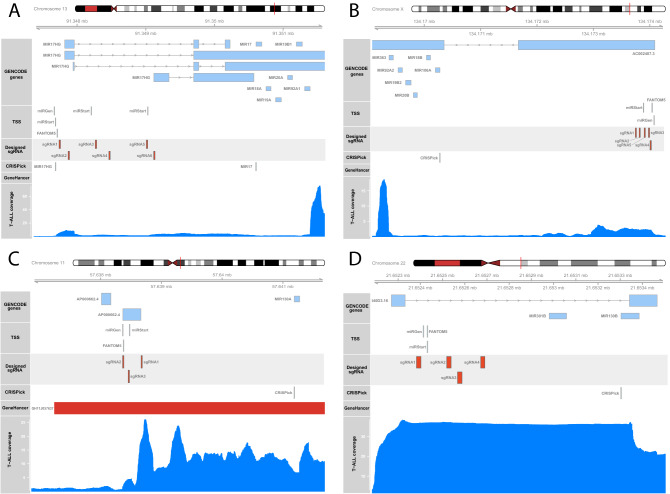
The location of genomic regions targeted by sgRNAs designed to repress the expression of miRNAs. The GENCODE Genes section shows the location of genes and miRNAs in regions of interest. The TSS section shows the location of putative TSSs predicted by miRStart, FANTOM5 and DIANA miRGen v.3 tools. The Designed sgRNAs section shows the positions recognized by sgRNAs tested in the study. The CRISPick section shows the TSSs predicted by CRISPick tool. The GeneHancer section shows the presence of enhancer sequences in the regions of interest, derived from GeneHancer database. The T-ALL Coverage section shows the coverage of reads from RNA-seq data for 64 T-ALL samples mapping to the region of interest. TSS prediction for: (**A**) miRNA cluster mir-17–92; (**B**) miRNA cluster mir-106a-363; (**C**) mir-130a; (**D**) mir-130b.

In order to identify TSS regions for each pri-miRNA transcript, we applied three online tools dedicated specifically for miRNA TSS prediction: miRStart^32^, FANTOM 5 miRNA atlas^33^, and DIANA-miRGen v.3^34^, which all integrate data from small RNA sequencing experiments. For comparison, we used CRISPick tool (not miRNA-dedicated), which enables TSS identification and sgRNA design for CRISPRi/CRISPRa in a single step. In Fig. 2 we show the putative TSSs of each targeted miRNA/miRNA cluster locus, predicted by the three miRNA-dedicated tools and by the CRISPick tool. Strikingly, in all analyzed cases the TSS location predicted by CRISPick was noticeably different than the predictions of miRNA-dedicated tools. To determine which prediction is more accurate, we utilized RNA-seq data of 64 pediatric T-cell acute lymphoblastic leukemia (T-ALL) samples [data unpublished], analyzed as described previously^35^. We show that for almost all TSSs, the read coverage in the regions predicted by miRNA-dedicated tools is noticeably higher than in putative TSSs indicated by CRISPick tool. The only exception was mir-130b locus, characterized by a uniform coverage across the whole analyzed region (Fig. 2D). Thus, for sgRNA design we focused on TSSs predicted by miRStart, FANTOM5 and DIANA-miRGen.

For mir-17–92 cluster, we chose three potential TSS regions predicted by miRStart, one of which was in close proximity to the TSS predicted by FANTOM 5 miRNA atlas and DIANA-miRGen. The remaining two potential TSS were predicted only by miRStart, but they were ranked higher than the one that was similarly predicted by all three tools. Additionaly, the highly ranked TSS in miRStart showed the presence of expression sequence tag (EST), which strongly supports the transcriptional activity of this locus. For all predicted TSSs, two sgRNAs were designed (Fig. 2A).

In case of mir-106a-363 cluster, the most likely TSS predicted by miRStart was selected. The distance between this region and potential TSSs predicted by FANTOM 5 miRNA atlas and by DIANA-miRGen were only 151 bp and 188 bp, respectively. Five sgRNAs were designed for this region (Fig. 2B).

For miR-130a-3p, the TSS predictions by all three tools were similar, with a maximum of 126 bp of difference. We designed three sgRNAs targeting this locus (Fig. 2C). For miR-130b-3p, the predictions of all three tools were very consistent, with up to 20 bp of difference. We designed four sgRNAs for the repression of this region (Fig. 2D).

### CRISPRi-mediated repression of paralogous clusters: mir-17–92 and mir-106a-363

We selected two cell lines, JURKAT and ALL-SIL, representing the same type of malignancy (T-ALL) as the patient samples used to determine the accuracy of TSS prediction tools, based on coverage in RNA-seq. Both cell lines have high endogenous expression of miRNAs encoded within studied miRNA clusters^36,37^. We transduced these cell lines to induce stable expression of dCas9-KRAB fusion protein (Supplementary Figs. S1–S5]. Next, the dCas9-KRAB expressing cells were transduced with vectors encoding each sgRNA targeting studied miRNA clusters. As a control, cells transduced with vector encoding non-targeting sgRNA were used.

**Figure 3:**
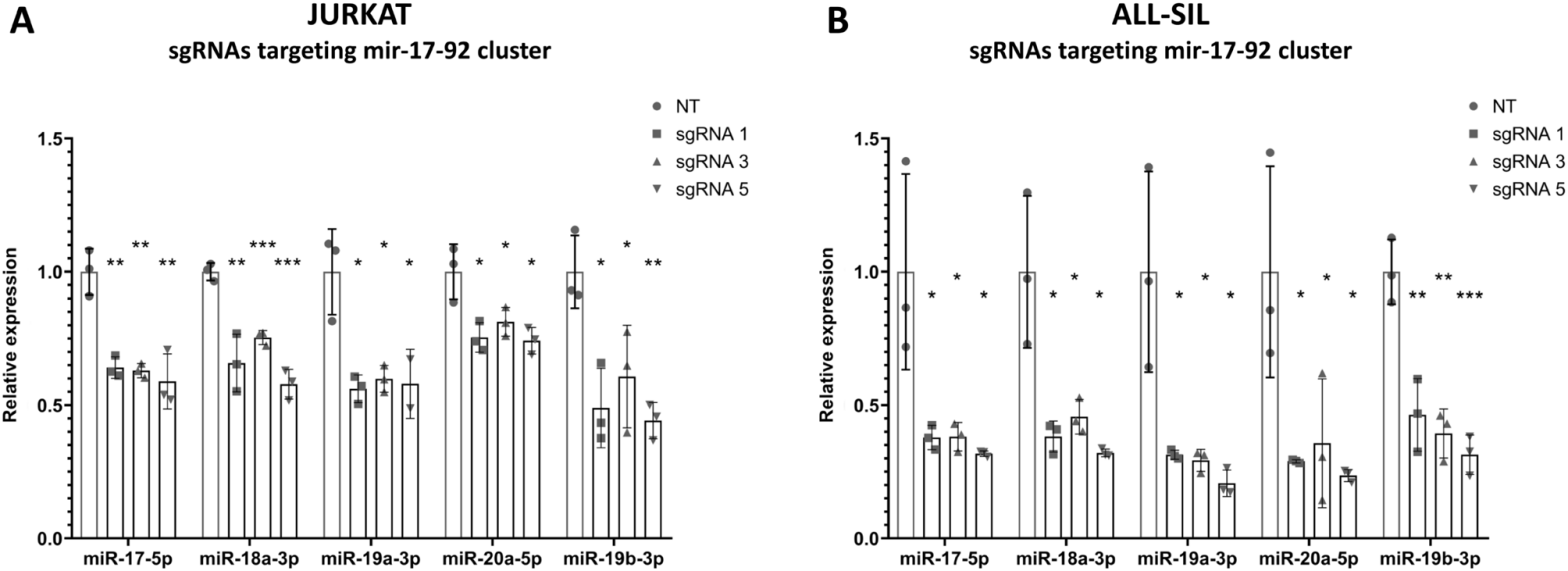
Expression of miRNAs encoded within mir-17–92 cluster upon dCas9-KRAB-mediated inhibition of this locus. (**A**) Normalized expression of miR-17-5p, miR-18a-3p, miR-19a-3p, miR-20a-5p and miR-19b-3p upon the use of three most effective sgRNAs targeting putative TSS of mir-17–92 cluster in JURKAT cell line. (**B**) Normalized expression of miR-17-5p, miR-18a-3p, miR-19a-3p, miR-20a-5p and miR-19b-3p upon the use of three most effective sgRNAs targeting putative TSS of mir-17–92 cluster in ALL-SIL cell line. Scr—scrambled control. *p < 0.05; **p < 0.01; ***p < 0.001.

After 14 days of culture, the cells were harvested and the expression of target miRNAs was assessed via RT-qPCR. In case of mir-17–92 cluster, three out of six sgRNAs appeared to effectively repress the expression of all clustered miRNAs (miR-17-5p, miR-18a-3p, miR-19a-3p, miR-20a-5p, miR-19b-3p). The observed repression effect was more profound in ALL-SIL cell line (Fig. 3B) than in JURKAT cell line (Fig. 3A). We also demonstrated that the expression of three miRNAs (miR-20b-5p, miR-106a-5p, miR-106b-5p) from paralogous miRNA clusters (mir-106a-363 and mir-106b-25) with almost identical sequence to miR-17-5p and miR-20a-5p (Supplementary Table S1) was not decreased upon inhibition of mir-17–92 cluster (Supplementary Fig. S6). Thus, dCas9-KRAB mediated repression proved highly specific, with no off-target inhibition of the analyzed miRNAs with high sequence homology. The exception was miR-20b-5p in ALL-SIL cells, which expression slightly decreased upon the use of two out of three sgRNAs targeting mir-17–92 cluster (Supplementary Fig. S6F). Of note, in some cases the expression of miRNAs with high sequence homology to miRNA encoded within the repressed cluster increased, suggesting the existence of compensatory mechanisms (e.g. miR-106b-5p in both cell lines) (Supplementary Fig. S6C, D).

**Figure 4:**
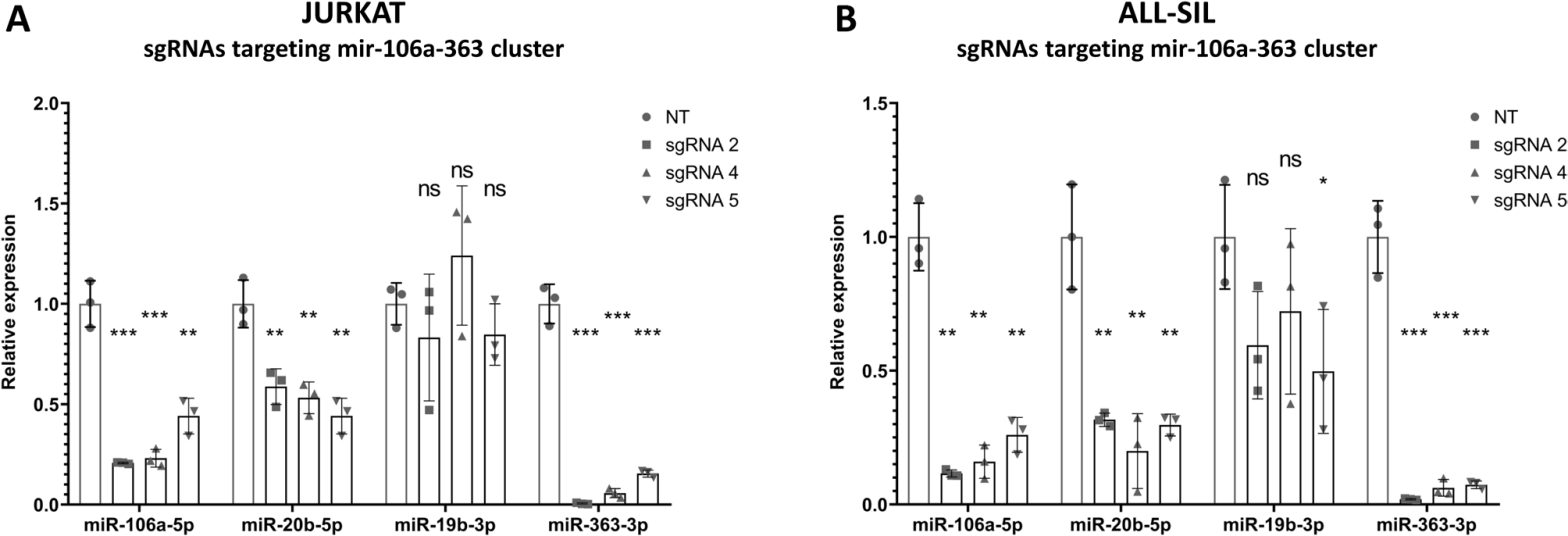
Expression of miRNAs encoded within mir-106a-363 cluster upon dCas9-KRAB-mediated inhibition of this locus. (**A**) Normalized expression of miR-106a-5p, miR-20b-5p, miR-19b-3p and miR-363-3p upon the use of three most effective sgRNAs targeting putative TSS of mir-106a-363 cluster in JURKAT cell line. (**B**) Normalized expression of miR-106a-5p, miR-20b-5p, miR-19b-3p and miR-363-3p upon the use of three most effective sgRNAs targeting putative TSS of mir-106a-363 cluster in ALL-SIL cell line. Scr—scrambled control. *p < 0.05; **p < 0.01; ***p < 0.001.

Our approach was also highly effective in case of the second studied miRNA cluster, mir-106a-363. Three out of five tested sgRNAs showed the satisfactory effectiveness. Three miRNAs from this cluster (miR-106a-5p, miR-20b-5p, miR-363-3p) were significantly repressed in both cell lines (Fig. 4A, B). The expression of miR-19b-3p was significantly decreased only in ALL-SIL cell line upon the use of one out of three sgRNAs (Fig. 4B). This can be explained by the fact that mature miR-19b-3p is also expressed from mir-17–92 cluster locus (Supplementary Table S1). Hence, silencing only one of these two loci may not be sufficient to repress this miRNA. Importantly, dCas9-KRAB inhibition of mir-106a-363 locus did not lead to off-target inhibition of miRNAs with high sequence homology from paralogous clusters (miR-17-5p, miR-106b-5p, miR-20a-5p) (Supplementary Fig. S7), with the exception of miR-17-5p (affected by two sgRNAs) and miR-20a-5p (affected by 1 sgRNA) in ALL-SIL cell line only (Supplementary Fig. S7B, F). Again, we also observed an increased expression of one of the studied miRNAs (miR-106b-5p) upon repression of mir-106a-363 cluster.

### Comparison of CRISPRi and si/shRNA-mediated repression of highly similar miRNAs

We aimed to compare CRISPRi (inhibiting transcription of miRNA coding sequences at genomic level) with broadly used siRNA/shRNA silencing approaches (inhibiting mature miRNA transcript) in terms of the effectiveness and specificity towards miRNAs with high sequence homology. We evaluated the expression of miR-20b-5p and miR-20a-5p from a paralogous cluster, upon inhibition of miR-20b-5p via transient transfection with synthetic miRNA inhibitor and via stable transduction with miRZip miRNA knockdown vector in DND-41 cell line. Both, the transfection with miR-20b-5p inhibitor (Supplementary Fig. S8A) and tranduction with miRZip shRNA vector targeting miR-20b-5p (Supplementary Fig. S8B) resulted in effective blocking of target miRNA but also of miRNA with high sequence homology (miR-20a-5p).

**Figure 5:**
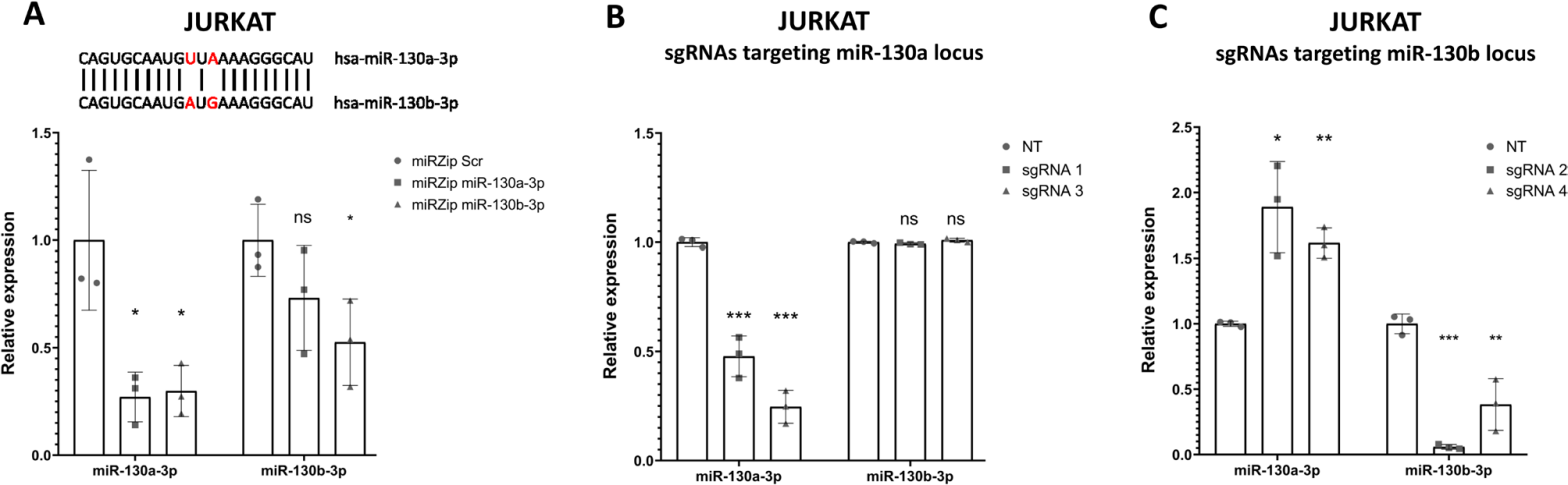
Comparison of specificity of miRZip and dCas9-KRAB approaches towards selective silencing of miR-130a-3p and miR-130b-3p. (**A**) Normalized expression of miR-130a-3p and miR-130b-3p upon the use of miRZip vector encoding shRNAs targeting miR-130a-3p or miR-130b-3p as compared do scrambled control miRZip vector (Scr) in JURKAT cell line. (**B**) Normalized expression of miR-130a-3p and miR-130b-3p upon the use of dCas9-KRAB system and sgRNAs targeting mir-130a TSS as compared do scrambled control (Scr) in JURKAT cell line. (**C**) Normalized expression of miR-130a-3p and miR-130b-3p upon the use of dCas9-KRAB system and sgRNAs targeting mir-130a TSS as compared do scrambled control (Scr) in JURKAT cell line. *p < 0.05; **p < 0.01; ***p < 0.001.

Next, we compared the effectiveness and specificity of CRISPRi and shRNA-inhibition towards miRNAs with high sequence homology, but not encoded in paralogous clusters. We analyzed the expression of two miRNAs, hsa-miR-130a-3p and hsa-miR-130b-3p, (encoded on chromosomes 11 and 22, respectively) in JURKAT and ALL-SIL cell lines, upon the use of CRISPRi as compared to shRNA-mediated repression (using miRZip miRNA knockdown vector, targeting hsa-miR-130a-3p and hsa-miR-130b-3p). We showed via RT-qPCR that miRZip shRNAs effectively block target miRNAs but they lack specificity—the level of hsa-miR-130a-3p is simultaneously decreased upon inhibition of hsa-miR-130b-3p. Moreover, the off-target inhibition of hsa-miR-130a-3p is even stronger than the inhibition of target hsa-miR-130b-3p (Fig. 5A).

In contrast, two sgRNAs designed to target hsa-miR-130a-3p locus effectively repressed miRNA in both cell lines, with no off-target repression of hsa-miR-130b-3p, demonstrating high specificity of CRISPRi (Fig. 5B; Supplementary Fig. S9A). Similarly, the use of two most effective sgRNAs targeting hsa-miR-130b-3p locus specifically repressed hsa-miR-130b-3p, with no repression of hsa-miR-130a-3p (Fig. 5C; Supplementary Fig. S9B). Again we observed increased expression of miR-130a-3p upon repression of miR-130b-3p, which may be indicative of the existence of compensatory mechanisms.

Using these examples we demonstrated that siRNA/shRNA approaches lack specificity towards targeted miRNAs. Since it is not possible to distinguish phenotypic effects of the simultaneous off-target inhibition of highly similar miRNAs, using such unspecific approaches might bias the results of experiments aimed at elucidating miRNA roles in a given biological context.

### The effect of CRISPRi-mediated miRNA repression on the expression of target genes

To further assess the effectiveness of CRISPRi-mediated repression of miRNAs, we evaluated the level of selected target genes for miRNAs of interest. We chose *BCL2L11* (also known as *BIM*), which we have previously shown as a common target for miR-20b-5p and miR-363-3p in T-ALL cells in vitro^38^. Since *BCL2L11* can potentially be targeted by miRNAs from miR-17, miR-19 and miR-92 seed families, we analyzed its expression upon CRISPRi-mediated silencing of mir-106a-363 cluster (Supplementary Figs. S10–S12). All three sgRNAs targeting this cluster resulted in significant increase of *BCL2L11* mRNA level, providing evidence for an effective inhibition of these miRNAs by CRISPRi (Supplementary Fig. S10C). This effect was also observable at the protein level of BCL2L11 (Supplementary Fig. S10D). Similarly, CRISPRi-mediated repression of miR-130a-3p and miR-130b-3p resulted in an increased level of *PTPRG*, being the common target gene for both these miRNAs (Supplementary Fig. S10A, B).

## Discussion

Here we present the approach for the identification of putative TSSs of miRNAs using complementary on-line tools (miRStart, FANTOM 5 and DIANA miRGen), followed by designing and testing of several sgRNAs per each selected TSS. To the best of our knowledge, our study is the first to provide direct comparison of the specificity of miRNA repression using dCas9-KRAB and a method utilizing siRNA/shRNA. We demonstrated several advantages offered by this approach over siRNA/shRNA. We propose a strategy (Fig. 1) and recommendations on how to use CRISPR/dCas in miRNA studies and how to overcome the challenges: an accurate prediction of miRNA TSS and the design of effective sgRNAs.

The first step is the identification of putative TSS for miRNAs of interest based on several (here—three) online tools and manual comparison of locations of TSSs predicted by these resources. We have demonstrated, using RNA-seq data from a large group of T-ALL patients’ samples, that TSS predictions by miRNA-dedicated tools are more accurate than TSS predictions by a standard tool (CRISPick). Thus, whenever possible, we recommend to analyze the coverage of reads mapping to the putative TSS region in the RNA-seq data of the cell line to be used or the representative cell type of interest, using own data or databases. If the overlap between miRStart, FANTOM 5 and DIANA miRGen is not conclusive, we suggest to select the TSS prediction tool which provides TSS location overlapping with high RNA-seq read coverage. The most probable TSS region, flanked by 50 bp upstream and 300 bp downstream from putative TSS sites, as recommended by Radzisheuskaya et al.^39^ should be used for sgRNA design. To this aim, we advise CRISPOR tool. We recommend to design 3–6 sgRNAs for each individual miRNA or miRNA cluster and to test them via assessment of the expression of miRNAs of interest in RT-qPCR upon the use of designed sgRNAs as compared to scrambled control.

The online resources we applied (miRStart, FANTOM 5 and DIANA-miRGen v.3) facilitate the prediction of regulatory regions for miRNAs: promoters, TSSs and binding sites for transcription factors. miRStart tool integrates data from high-throughput platforms: Cap Analysis of Gene Expression (CAGE), TSS Seq tags and H3K4me3 enrichment^32^. It also includes the information about the presence of expressed sequence tag (EST) and considers the conservation of putative TSS region between different vertebrate species^32^. FANTOM 5 miRNA atlas collects miRNA expression data from human tissues and putative miRNA promoters and TSS data, based on small RNA sequencing data and CAGE experiments^33^. DIANA-miRGen v.3 predicts miRNA TSSs by integrating data from RNA sequencing and H3K4me3 ChIP-seq with RNA polymerase II occupancy data and DNase-Seq, which enables to identify the open chromatin regions^34,40^. Additionally, based on our RNA-seq data we supplemented TSS prediction with the information on the coverage of reads mapping to the loci of interest. This in silico analysis enabled us to confirm that these genomic regions are actively transcribed in the cells of interest and the presence of peaks in the proximity of predicted TSS suggests that these are indeed the sites where pri-miRNA transcription initiates.

In case of silencing mir-17–92 cluster, we designed sgRNAs targeting three putative TSS sequences, which were several hundred base pairs apart from one another. These genomic regions were characterized by the presence of read coverage peak in our RNA-seq results. Surprisingly, targeting each of these regions turned out to be effective for the repression of clustered miRNAs. It is possible that this miRNA cluster may have more than one alternative TSSs^41^. Other possible explanation is that the CRISPRi utilizing KRAB domain, may be effective even in case of not entirely precise prediction of a putative TSS. This is because CRISPRi, unlike classical CRISPR-Cas9 system, relies on local heterochromatinization and not on precise cutting of target region. The KRAB-mediated heterochromatin formation can range up to several thousand base pairs from the DNA region directly bound to KRAB domain^42^. Nevertheless, we recommend designing and testing multiple sgRNAs for putative TSS regions predicted by several tools, to select sgRNAs which provide effective modulation of miRNA expression.

There is currently a wide range of tools for sgRNA design, as reviewed by Sledzinski et al.^43^. Among them, CRISPick^44,45^ and CRISPR-ERA^46^ offer the identification of TSS and sgRNA design in a single step. These tools are highly useful for CRISPRa/CRISPRi of protein coding genes. Yet, due to incomplete annotation of miRNA TSSs they are not optimal for miRNA studies. As we have shown (Fig. 2), TSS predictions by CRISPick, in case of the studied miRNAs were strikingly distant from those predicted by miRNA-dedicated tools. In our approach we used CRISPOR tool for sgRNA design, which is considered as one of the most accurate and versatile tools for sgRNA design^43^. CRISPOR employs several predictive scoring models for the assessment of sgRNA effectiveness and potential off-target activity. It also provides information facilitating the cloning procedure and in vitro expression^47^.

We have demonstrated that CRISPRi outperforms si/shRNA-based miRNA repression in terms of specificity, as we have shown for miRNAs with high sequence homology: hsa-miR-130a-3p versus hsa-miR-130b-3p (Fig. 5A) and hsa-miR-20b-5p versus hsa-miR-20a-5p (Supplementary Fig. S8). The approach we propose, by targeting miRNAs at the genomic level, enable to selectively repress miRNAs of interest, without repression of other miRNAs with high sequence homology. Of note, CRISPRi-mediated repression does not lead to full abolishment of the mature miRNA transcript as in case of the use of synthetic miRNA inhibitor molecules. The effectiveness of silencing may vary from 30 to 90%. However, this moderate silencing can be of benefit, especially in case of studying the role of miRNAs overexpressed in diseases. For instance, miRNAs targeted in our study (e.g. hsa-miR-20b-5p and hsa-miR-20a-5p) are overexpressed in T-cell acute lymphoblastic leukemia as compared to normal T-cells, as we have described previously^37^. Although the expression level of these miRNAs is higher in cancer cells than in their normal counterparts, still they are present in nonmalignant cells, since they are needed for their normal physiological function. If we aim to distinguish between the effect of their overexpression in a disease state from the effect of their normal physiological expression, the less dramatic CRISPRi-mediated decrease of miRNA expression may provide more reliable information. As an example, we show in Supplementary Fig. S13 the expression of miR-20b-5p and miR-363-3p in Jurkat and ALL-SIL cell lines upon the use of dCas9-KRAB and sgRNA4 targeting these miRNAs as compared to expression levels of these miRNAs in normal T-cells. We show that the use of CRISPRi enables to decrease the expression of these miRNAs to the level comparable to that of healthy cells, thus providing more physiological conditions.

Additionally we show, for several pairs of almost identical miRNAs encoded in different loci, that repression of one miRNA may result in an increased expression of homologous miRNAs from the same seed family. Such phenomenon may suggest the existence of mechanisms, leading to the upregulation of miRNAs potentially cooperating in the regulation of a given biological process, to compensate for the loss of function of a repressed miRNA. These mechanisms might naturally contribute to phenotypic effects observed in a given experiment, and should be considered while concluding the experiment results. Of note, such compensatory mechanisms would not be even observable, when inhibiting mature miRNAs. This highlights another advantage of CRISPRi over si/shRNA.

The proposed CRISPRi approach may also be adopted for repressing individual miRNA alleles. To this aim the sgRNA should be designed in the regions containing SNPs specific for one of the alleles. In consequence, the dCas9-KRAB complex should target TSS of only one of the alleles resulting in a monoallelic repression of studied miRNA. This approach would be useful e.g. for studying the consequences of monoallelic mutations affecting mature miRNA sequence and its effect on target gene binding, which may play a role e.g. in cancer^48^.

## Limitations of the study

The CRISPRi-based strategy for miRNA repression has a few drawbacks that should be considered at the step of study design. CRISPRi may not be an optimal choice in case of miRNAs arising in non-canonical biogenesis pathways, e.g. intronic miRNAs that do not have their own TSS and thus their transcription cannot be targeted by the dCas9-KRAB complex. Moreover, as shown recently, pri-miRNAs may function as lncRNAs and act their own biological roles, independently from mature miRNAs they give rise to. In such cases, inhibition of miRNAs on the genomic level using CRISPRi, may result in unexpected events related to disturbance of lnc-pri-miRNAs. If the research aims to distinguish the roles of miRNAs themselves from the roles of their lnc-pri-miRNA transcripts, the experiment needs to include the comparison of phenotypic effects of CRISPRi repression with the effects of targeting mature miRNAs. Moreover, the genomic loci of TSS for miRNAs may contain some regulatory elements, e.g. transcription enhancers; as TSS region for has-mir-130a in this study (Fig. 2C). In such cases, CRISPRi-mediated inhibition may potentially result in off-target effects on the expression of genes related to this enhancer. Thus, the method of miRNA modulation should be carefully selected and adjusted to the scientific question that the research is aimed to answer.

## Conclusion

The proposed approach, including the use of several tools for TSS prediction combined with RNA-seq analysis and thorough testing of designed sgRNAs, enables effective and precise repression of highly similar miRNAs and miRNA clusters. We demonstrate the specificity of CRISPRi approach as compared to methods based on si/sh-RNA-mediated repression (transient transfection with synthetic miRNA inhibitor and transduction with shRNA coding vector targeting mature miRNA). We also highlight that CRISPRi might reveal the existence of compensatory mechanisms related to inhibition of miRNA with high sequence homology, and by the proper selection of sgRNAs might enable to control for their influence on the experiment. We postulate that CRISPR/dCas9-based techniques are an attractive solution to overcome important challenges in the experiments aimed at manipulation of miRNA expression.

## Materials and methods

### Cell lines, cell culture and normal T-cell samples

The HEK293T cell line was a kind gift from Prof. Maciej Kurpisz lab (Institute of Human Genetics, Polish Academy of Sciences, Poland). Cells were cultured under standard conditions in Dulbecco’s modified Eagle’s medium (Gibco, Thermo Fisher Scientific, Waltham, MA, USA) with 10% fetal bovine serum (Gibco, Thermo Fisher Scientific) and 1% penicillin/streptomycin solution (Sigma Aldrich, St. Louis, MO, USA). DND-41 and Jurkat T-cell acute lymphoblastic leukemia cell lines were purchased from the Leibniz Institute DSMZ—German Collection of Microorganisms and Cell Cultures. ALL-SIL T-cell acute lymphoblastic leukemia cell line was a kind gift from Prof. Pieter Van Vlierberghe lab (Cancer Research Institute Ghent (CRIG), Belgium). Cells were cultured under standard conditions in RPMI-1640 medium (Gibco, Thermo Fisher Scientific) with 10% (for Jurkat and DND-41) or 20% (for ALL-SIL) of fetal bovine serum (Gibco, Thermo Fisher Scientific). Normal T-cell samples were obtained as described previously^37^.

### sgRNA and shRNA design

TSS for studied miRNAs were predicted with the use of three online tools: miRStart^32^, FANTOM 5 miRNA atlas^33^ and DIANA-miRGen v.3^34^. All the most highly ranked potential TSSs predicted by these tools were included in further steps of sgRNA design. Next, CRISPOR online tool^47^ was utilized to design sgRNAs targeting the region − 50 to + 300 bp from the predicted, most probable TSS. We utilized GRCh37 version of human genome and canonical NGG PAM sequence for *Streptococcus pyogenes* Cas9 (SpCas9) protein. 3–6 sgRNAs/predicted TSS, proposed by CRISPOR were selected according to their specificity score and predicted efficiency. The shRNA targeting mature sequences of miRNA of interest were designed according to Yang et al.^49^ and System Biosciences (Palo Alto, CA, USA) guidelines.

The sgRNA and shRNA coding sequences were purchased in a form of DNA oligonucleotides from Genomed (Warsaw, Poland) and cloned into appropriate expression vector. The list of sgRNA and shRNA oligonucleotides is shown in Supplementary Table S2.

### Expression vectors

For expression of shRNA targeting mature miRNA, miRZip knockdown system (System Biosciences) was used. Constructs were cloned into a pGreenPuro lentiviral vector (System Biosciences). As a control for shRNA experiments pGreenPuro scrambled vector (System Biosciences) was used. pGreenPuro scrambled control vector and empty backbone vector were a kind gift from prof. Anke van den Berg and dr. Joost Kluiver^13^.

For expression of dCas9-KRAB system, Lenti-dCas9-KRAB-blast vector was used^50^. For expression of sgRNA, pU6-sgRNA-Ef1alpha-Puro-T2A-GFP vector was used^51^. These vectors were a kind gift from Prof. Agnieszka Dzikiewicz-Krawczyk lab (Institute of Human Genetics, Polish Academy of Sciences, Poland). As a control for dCas9-KRAB experiments, pU6-sgRNA scrambled vector was used. This vector was a kind gift from Dr. Katarzyna Iżykowska (Institute of Human Genetics, Polish Academy of Sciences, Poland). The sequence of DNA oligonucleotides used for expression of scrambled sgRNA is shown in Supplementary Table S2. For lentiviral particles assembly, pRSV.REV, pMSCV-VSV-G and pMDLg/PRRE packing vectors were used.

### Transfection and lentiviral transduction

DND-41 cell line was transfected with negative control and hsa-miR-20b-5p inhibitors (miRVana, Thermo Fisher Scientific). Inhibitors were used in the final concentration of 100 nM. Cells were electroporated with the use of the Neon Electroporation System (Thermo Fisher Scientific) as described previously^38^.

For assembly of lentiviral particles, HEK293T cells were seeded on 6-well culture plate. Upon 70–80% confluence, the cells were transfected with 600 ng of each: pRSV.REV, pMSCV-VSV-G and pMDLg/PRRE packing vectors and 1200 ng of transfer vector. Transfection was performed with the use of JetPrime DNA/siRNA Transfection Kit (Polyplus Transfection, New York, NY, USA). After 24 h the transfection medium was replaced with 1 ml fresh medium. After 48 h the medium was collected and filtered with 0.45 µm filters.

For transduction of JURKAT and ALL-SIL cell lines, the target cells were seeded on 6-well plate in 1.8 ml of RPMI-1640 medium. 200 µl of filtered medium containing lentiviral particles was added to each well. For JURKAT and ALL-SIL cell lines, Polibrene Reagent (Sigma Aldrich, St. Louis, MO, USA) was added to each well at the final concentration of 8 µg/ml. For transduction of DND-41, cells were seeded on 12-well plate in 400 µl of RPMI-1640 medium. 400 µl of filtered medium containing lentiviral particles was added to each well. Additionally, 200 µl of TransDux Max Enhancer (System Biosciences) and 2 µl TransDux Reagent (System Biosciences) were added. Cells were spinfected for 90 min with the speed 1500 rpm at the temperature 32 °C. Antibiotic selection of transduced cells was started 3–4 days post transduction. For selection, puromycin dihydrochloride (Gibco, Thermo Fisher Scientific) or blasticidin (Thermo Fisher Scientific) were used at the concentration of 10 µg/ml. The selection procedure was conducted for 14 days. The effectiveness of transduction was assessed either by Western Blot for detection of Cas9 protein for Lenti-dCas9-KRAB-blast vector, or with the use of FlowSight flow cytometer for pU6-sgRNA-Ef1alpha-Puro-T2A-GFP vector (Luminex Corporation, Austin, TX, USA) with GFP as a marker.

### Western blot

10^7^ cells were lysed in 100 µl of RIPA buffer with protease inhibitor cocktail and EDTA (Thermo Fisher Scientific). Protein concentration was determined using Pierce BCA Protein Assay Kit (Thermo Fisher Scientific). Proteins were separated on 4–15% Mini-PROTEAN TGX Stain-free Gel (Bio Rad). After electrophoresis, proteins were transferred onto 0.45 µm PVDF Low Fluorescence membrane (Bio Rad, Hercules, CA, USA). Membranes were blocked using 5% non-fat milk and incubated with 1:2000 mouse anti-Cas9 antibody (#14697, Cell Signaling Technology, Danvers, MA, USA) or 1:2000 rabbit anti-BIM antibody (#2933, Cell Signaling Technology). After washing, membranes were incubated with horseradish peroxidase (HRP) conjugated with 1:10,000 anti-mouse (A9917, Sigma Aldrich) or 1:40,000 anti-rabbit (ab97051, Abcam, Cambridge, U) secondary antibody. Immunoreactive protein bands were detected with Clarity Western ECL Substrate for HRP (Bio-Rad) on Chemidoc Imaging System (Bio Rad). The abundance of target protein was assessed in reference to the total protein on a blot in Stain-Free technology using Image Lab 6.0.1 software (Bio Rad). Each experiment was conducted in three biological replicates.

### RNA extraction and RT-qPCR

The miRNeasy RNA isolation kit (Qiagen, Hilden, Germany) was used for the extraction of total RNA including the recovery of the small RNA fraction. RNA isolates were DNase treated and purified with use of RNA Clean and Concentrator Kit (Zymo Research, Irvine, CA, USA). RNA concentration was measured with Quantus Fluorometer (Promega, Madison, WI, USA) using Qubit HS RNA Assay Kit (Thermo Fisher Scientific). For miRNA quantification, total RNA was reverse transcribed with TaqMan Advanced miRNA cDNA Synthesis Kit (Thermo Fisher Scientific) according to the manufacturer’s protocol. TaqMan Fast Advanced Master Mix and predesigned TaqMan Advanced miRNA assays (Thermo Fisher Scientific) were used. Three endogenous control miRNAs (hsa-miR-16-5p, hsa-let-7a-5p and hsa-miR-25-3p) were selected using a strategy based on a comprehensive assessment of expression stability in our miRNA-seq data and in RT-qPCR, as previously described^52^. The full list of RT-qPCR miRNA assays is shown in Supplementary Table S3. For mRNA quantification, total RNA was reverse transcribed with iScript cDNA Synthesis Kit (Bio Rad) and HOT FIREPol EvaGreen qPCR Mix Plus (Solis Biodyne, Tartu, Estonia) was used. Geometric mean of *ACTB* and *GAPDH* expression was used for normalization of expression of the analyzed target genes. Primers were synthetized by Genomed (Warsaw, Poland). List of primers used for mRNA quantification is presented in Supplementary Table S2. All RT-qPCR analyses were conducted in two technical and three biological replicates with the use of 7900HT Fast Real-Time PCR System (Applied Biosystems, Foster City, CA, USA). Comparative deltaCT method (ΔΔCT) and Data Assist Software v. 3.01 (Thermo Fisher Scientific) were used for relative quantification of expression^53^.

### In silico analysis

Graphics showing sgRNA locations were created using Gviz Bioconductor library^54^, using Gencode gene annotations v38^55^, GeneHancer list of double-elite enhancers^56^ and mean coverage information obtained from our set of 64 T-ALL RNA-seq samples with 150 M reads per sample (data unpublished), analyzed as described previously^35^.

### Statistical analysis

For the comparison of two independent means, data were analyzed for normality with Shapiro–Wilk test and next the two groups were tested for equality of variances. Statistical significance of the results was calculated with unpaired two-tailed *t* test. All analyses and data visualization were performed with GraphPad Prism 8 software.

## Supplementary Information

Supplementary Information.
