# Supplementary material for: CRISPRi for specific inhibition of miRNA clusters and miRNAs with high sequence homology
Source: Sci Rep. 2022 Apr 15;12:6297. doi: 10.1038/s41598-022-10336-3 (PMC9012752; doi:10.1038/s41598-022-10336-3)
Supplement: Supplementary file 1 — Supplementary Information. [file 41598_2022_10336_MOESM1_ESM.pdf]

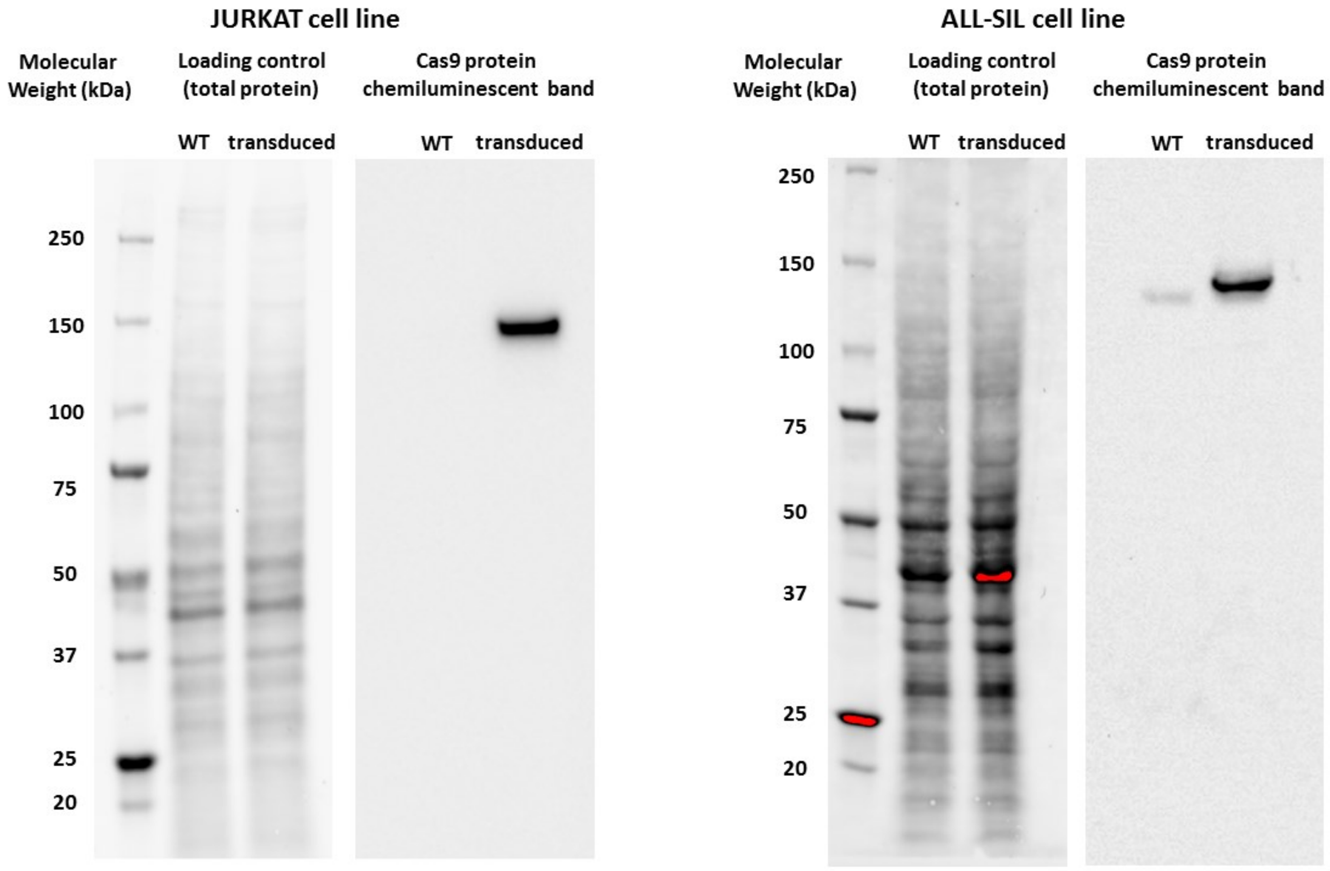

Supplementary Fig. 1 Western blots presenting the expression of Cas9 protein after transduction with dCas9-KRAB encoding vector in JURKAT and ALL-SIL cell lines. The left panels present the protein weight markers and total protein in the lane visualized via the stain-free technology, used as loading control. The right panels present chemiluminescent band after incubation with anti-Cas9 antibody. WT – wild type (non-transduced) cells; transduced – cells after transduction with dCas9-KRAB encoding vector.

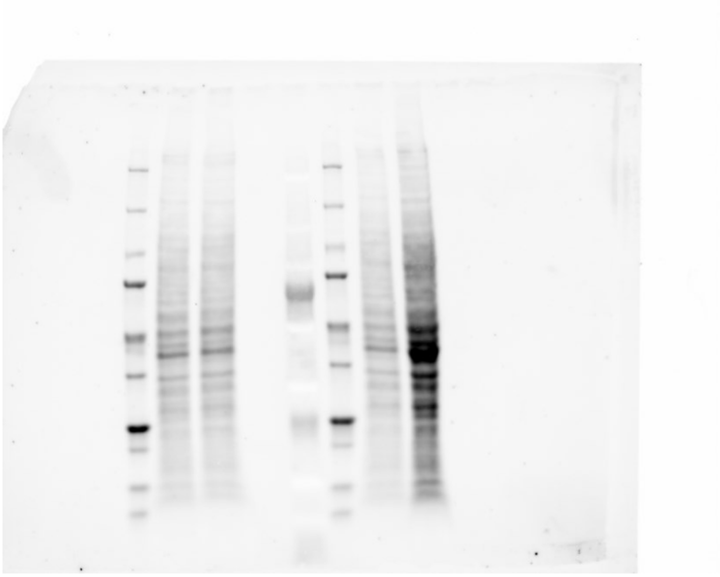

Supplementary Fig. 2 Uncropped stain-free total protein blot for ALL-SIL cell line (right side of the membrane), shown also on Supplementary Fig. 1A.

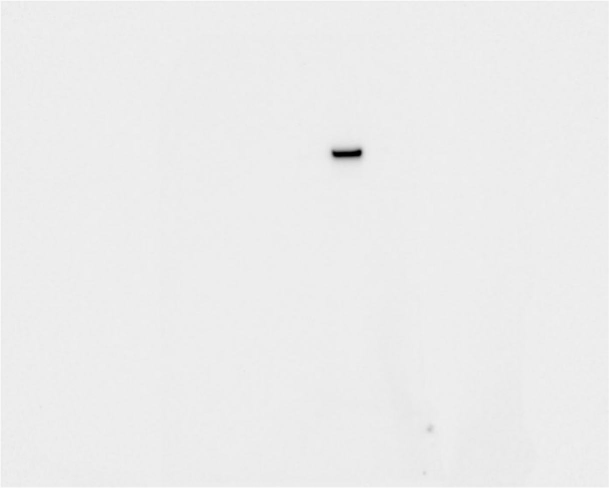

Supplementary Fig. 3 Uncropped chemiluminescent blot for JURKAT cell line (central part of the picture), shown also on Supplementary Fig. 1A.

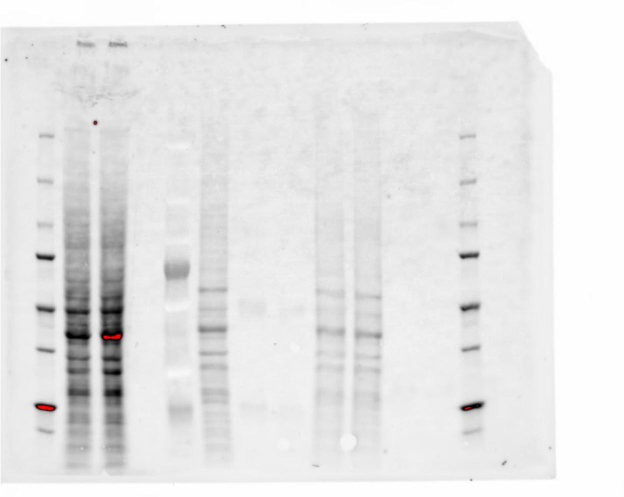

Supplementary Fig. 4 Uncropped stain-free total protein blot for ALL-SIL cell line (right side of the membrane), shown also on Supplementary Fig. 1B.

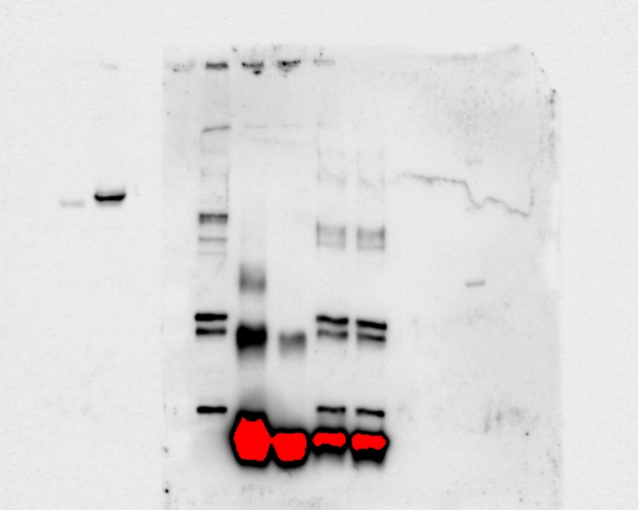

Supplementary Fig. 5 Uncropped chemiluminescent blot for ALL-SIL cell line(right side of the membrane), shown also on Supplementary Fig. 1B.

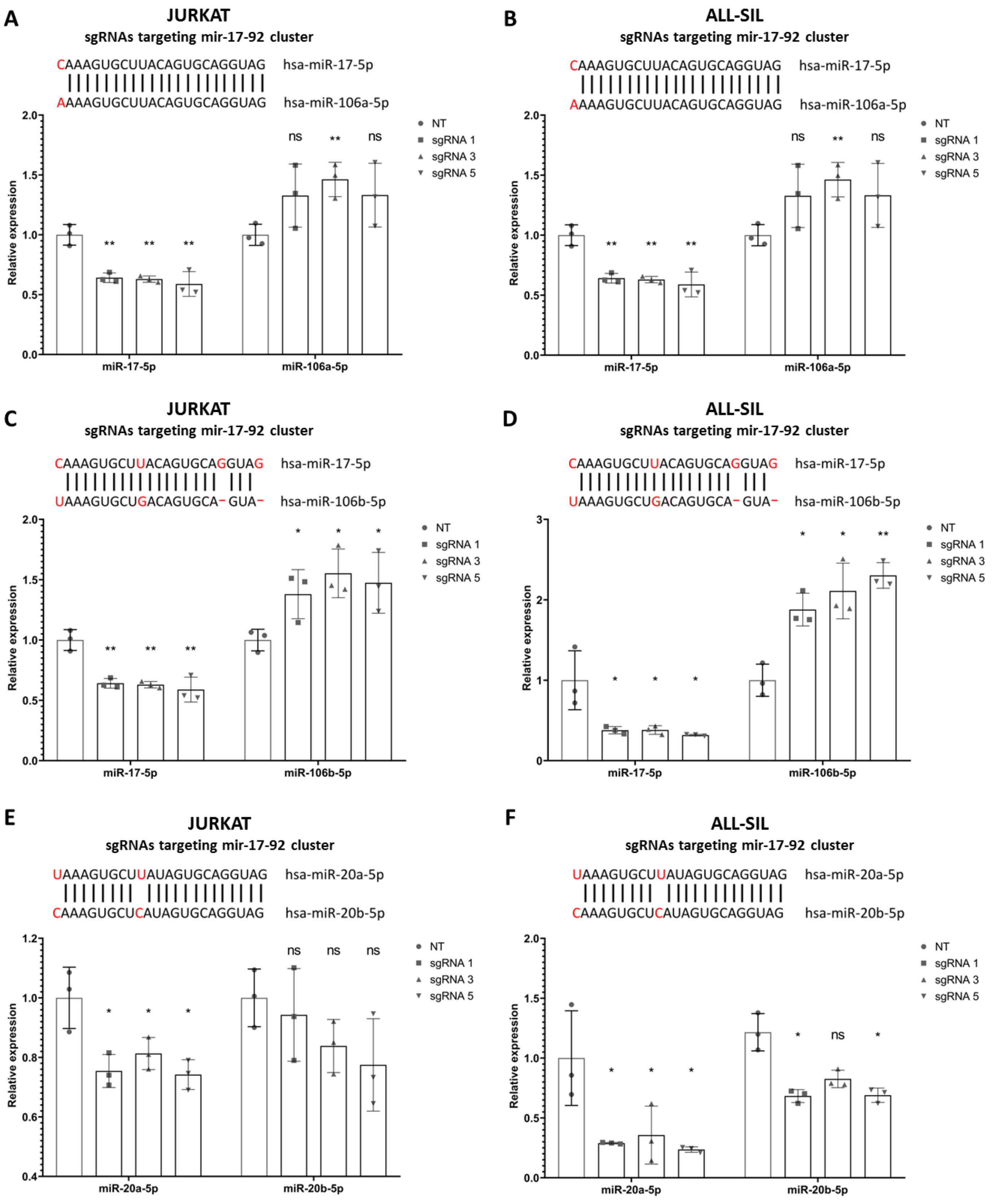

Supplementary Fig. 6 Comparison of expression of miRNAs from mir-17-92 cluster with miRNAs of high sequence homology, from mir-106a-363 and mir-106b-25 clusters, upon the use of three most effective sgRNAs targeting putative TSS of mir-17-92 cluster in JURKAT an ALL-SIL cell lines. Comparison of expression of: **A.** and **B.** miR-17-5p from mir-17-92 cluster and miR-106a-5p from mir-106a-363 cluster in JURKAT and ALL-SIL cell lines, respectively. **C.** and **D.** miR-17-5p from mir-17-92 cluster and homologous miR-106b-5p from mir-106b-25 cluster in JURKAT ALL-SIL cell lines, respectively. **E.** and **F.** miR-20a-5p from mir-17-92 cluster and homologous miR-20b-5p from mir-106a-363 cluster in JURKAT and ALL-SIL cell lines, respectively. NT – non-targeting control. * - p<0.05; ** - p<0.01; *** - p<0.001.

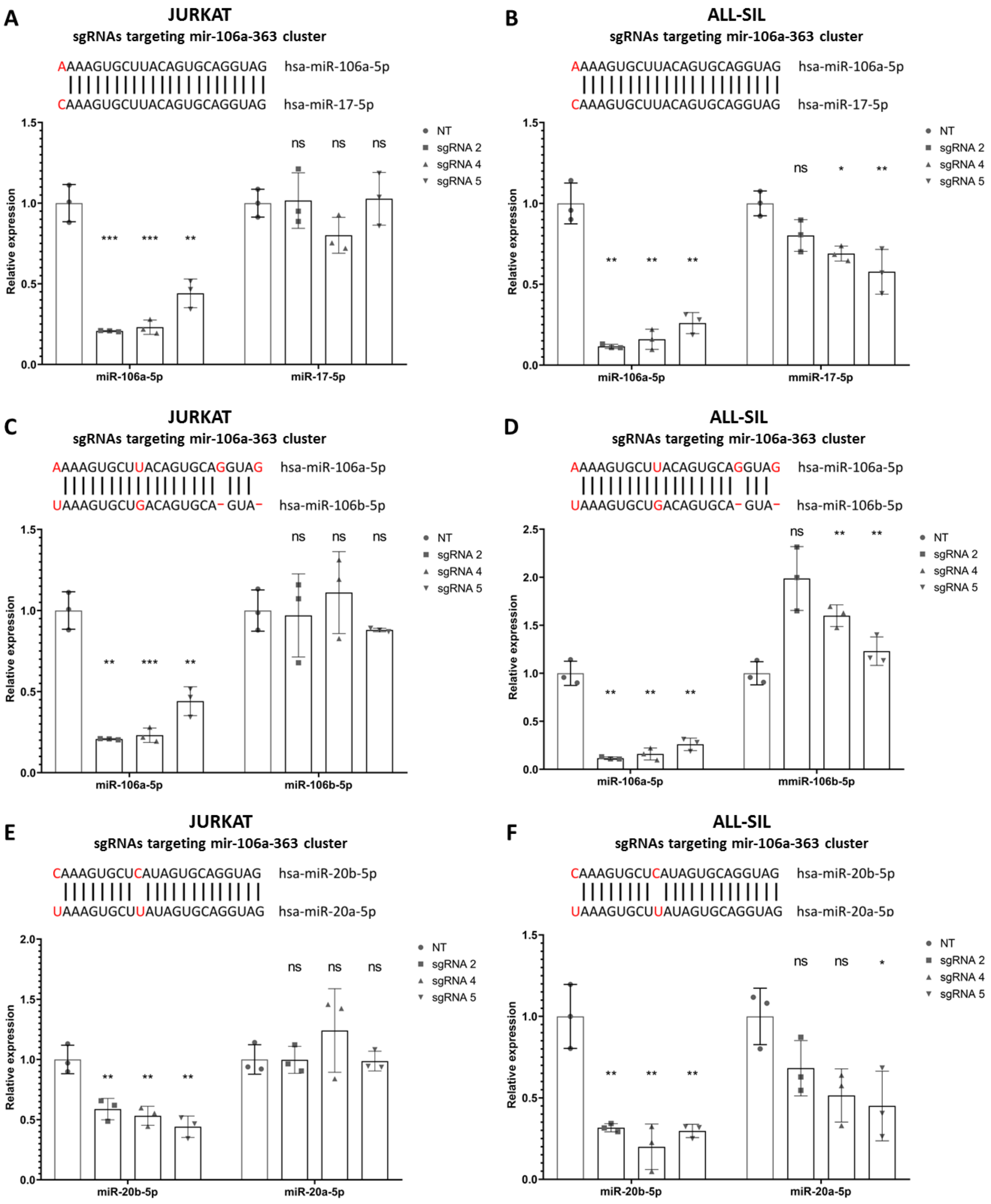

Supplementary Fig. 7 Comparison of expression of miRNAs from mir-106a-363 cluster with miRNAs of sequence homology, from mir-17-92 and mir-106b-25 clusters, upon the use of three most effective sgRNAs targeting putative TSS of mir-106a-363 cluster in JURKAT an ALL-SIL cell lines. Comparison of expression of: **A.** and **B.** miR-106a-5p from mir-106a-363 cluster and homologous miR-17-5p from mir-17-92 cluster in JURKAT and ALL-SIL cell lines, respectively. **C.** and **D.** miR-106a-5p from mir-106a-363 cluster and homologous miR-106b-5p from mir-106b-25 cluster in JURKAT and ALL-SIL cell lines, respectively. **E.** and **F.** miR-20b-5p from mir-106a-363 cluster and homologous miR-20a-5p from mir-17-92 cluster in JURKAT and ALL-SIL cell lines, respectively. NT – non-targeting control. * - p<0.05; ** - p<0.01; *** - p<0.001.

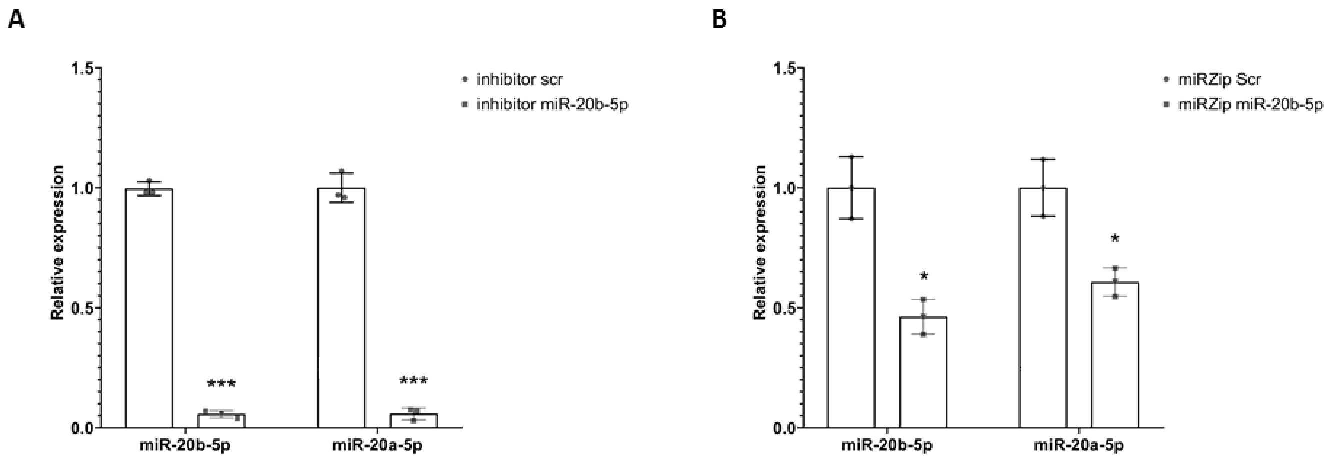

Supplementary Fig. 8 Comparison of specificity of transient transfection with miR-20b-5p synthetic miRNA inhibitor and transduction with miRZip shRNA targeting miR-20b-5p. **A.** Normalized expression of miR-20b-5p and miR-20a-5p upon the use of miRVana miRNA inhibitor targeting miR-20b-5p as compared do scrambled control inhibitor (Scr) in DND-41 cell line. **B.** Normalized expression of miR-20b-5p and miR-20a-5p upon the use of miRZip vector encoding shRNAs targeting miR-20b-5p as compared do scrambled control miRZip vector (Scr) in DND-41 cell line.* - p<0.05; ** - p<0.01; *** - p<0.001.

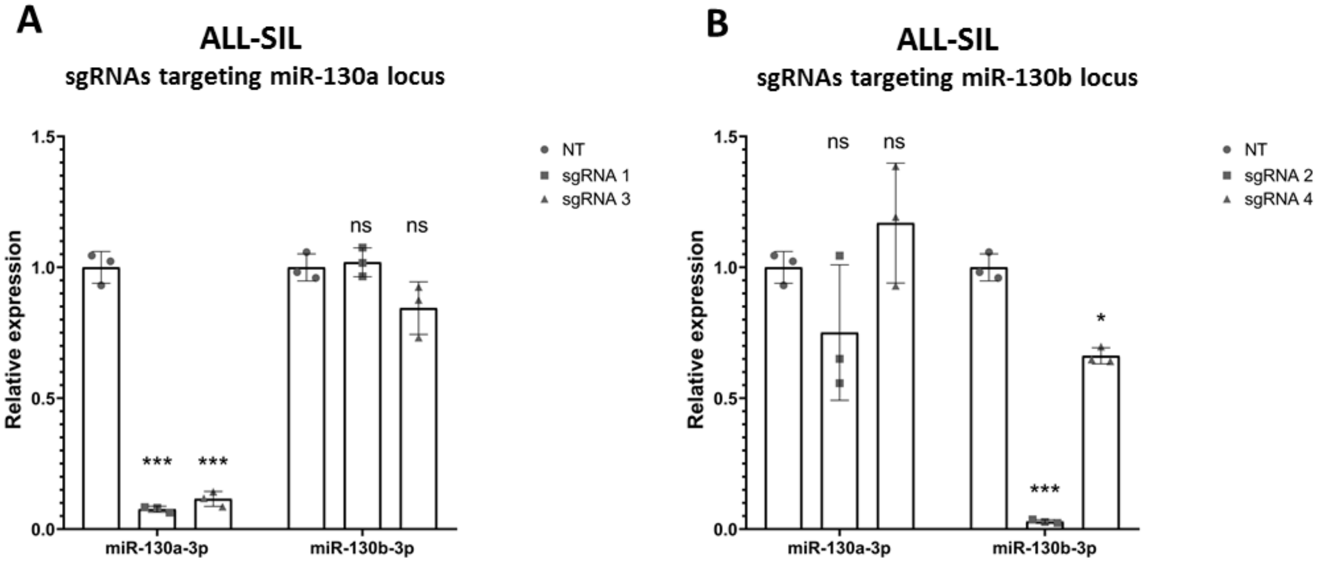

Supplementary Fig. 9 Effectiveness of dCas9-KRAB approach in selective silencing of miR-130a-3p and miR-130b-3p. **A.** Normalized expression of miR-130a-3p and miR-130b-3p upon the use of dCas9-KRAB system and sgRNAs targeting mir-130a TSS as compared do scrambled control (Scr) in ALL-SIL cell line. **B.** Normalized expression of miR-130a-3p and miR-130b-3p upon the use of dCas9-KRAB system and sgRNAs targeting mir-130a TSS as compared do non-targeting control (NT) in ALL-SIL cell line. * - p<0.05; ** - p<0.01; *** - p<0.001.

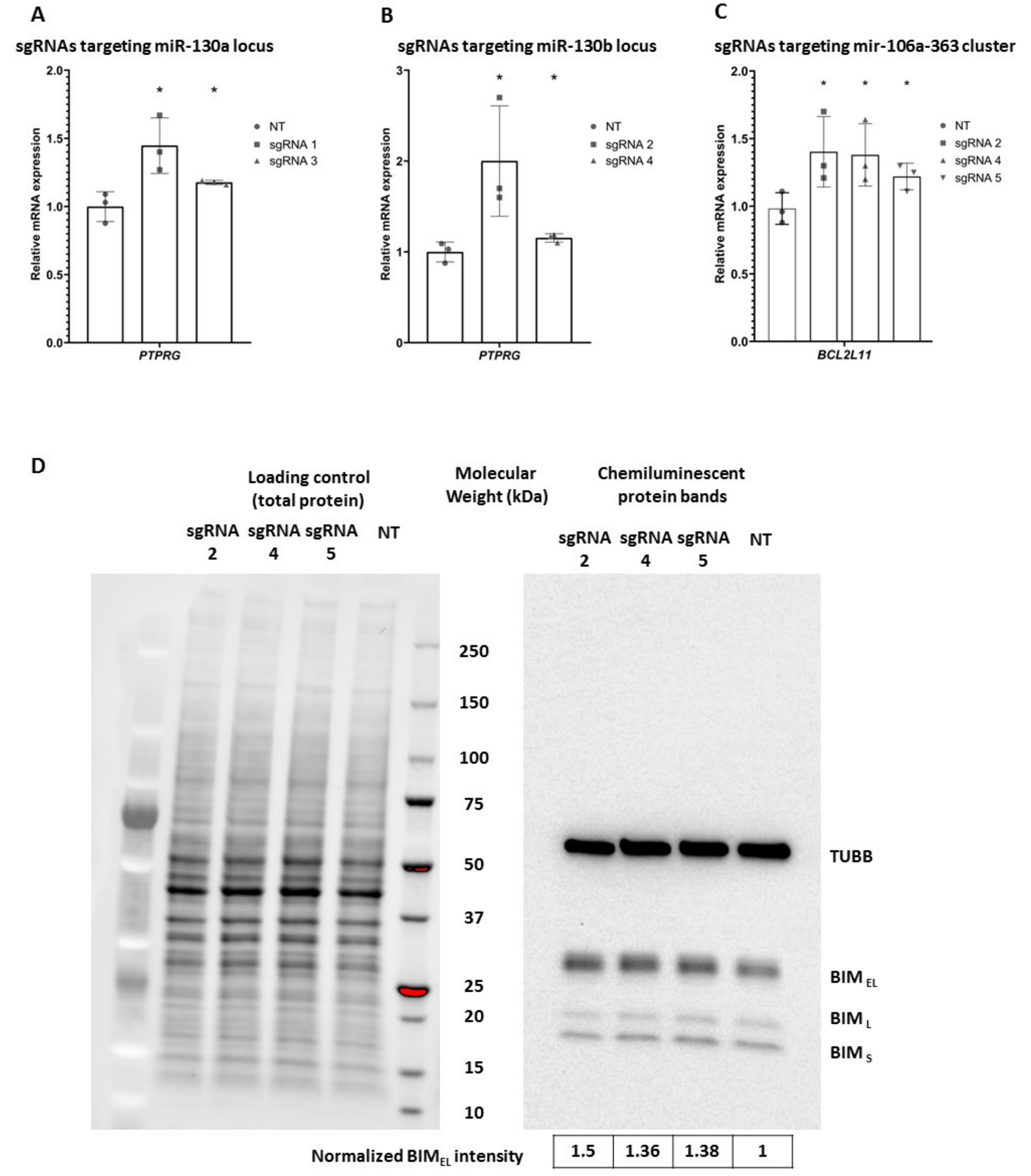

Supplementary Fig. 10 Evaluation of the expression of target genes for hsa-miR-130a-3p, miR-130b-3p and miRNAs from cluster mir-106a-363 upon CRISPRi mediated silencing of miRNAs of interest. Evaluation of mRNA level of *PTPRG* upon repression of **A.** miR-130a-3p or **B.** miR-130b-3p. **C.** Evaluation of mRNA level of *BIM* upon inhibition of miRNAs from mir-106a-363 cluster. **D.** Evaluation of protein level of BIM upon inhibition of miRNAs from mir-106a-363 cluster. The normalized BIM intensity was calculated as a ratio of intensity of band representing BIM protein to the intensity of band representing TUBB housekeeping protein. NT—non-targeting control; * $p < 0.05$.

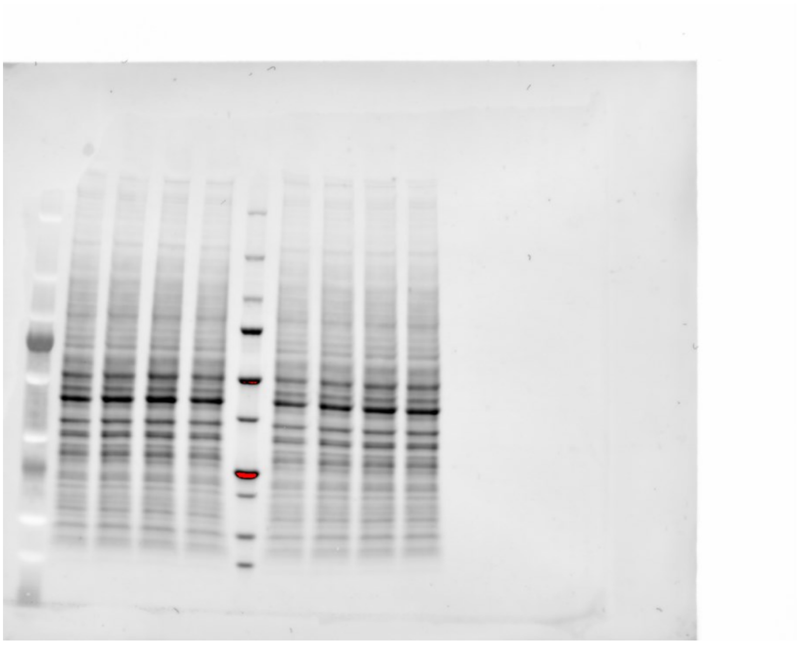

Supplementary Fig. 11 Uncropped stain-free total protein blot for ALL-SIL cell line (right side of the membrane), shown also on Supplementary Fig. 10D.

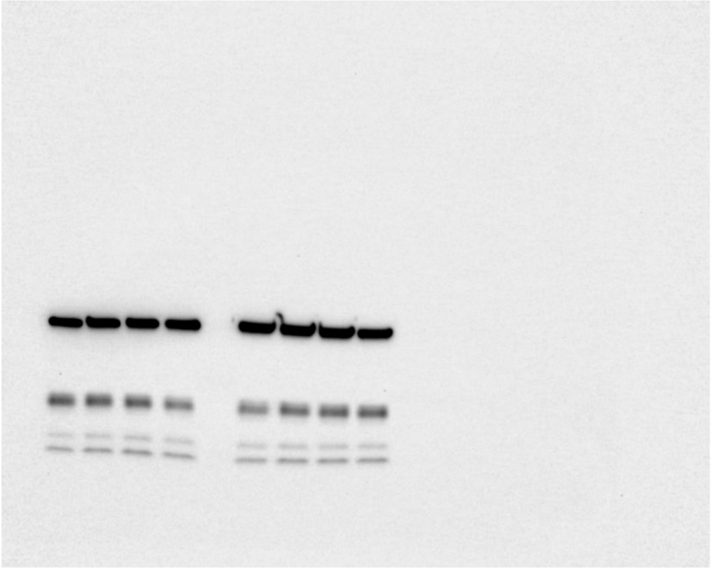

Supplementary Fig. 12 Uncropped chemiluminescent blot (right part of the picture), shown also on Supplementary Fig. 10D.

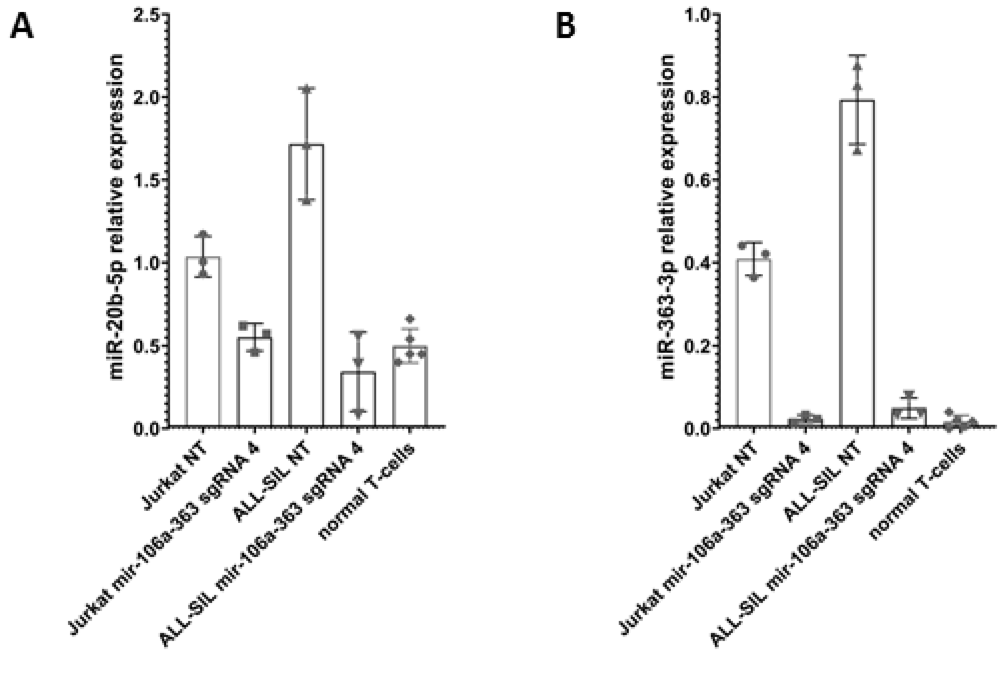

Supplementary Fig. 13 Evaluation of the expression of hsa-miR-20b-5p (**A.**) and hsa-miR-363-3p (**B.**), encoded in mir-106a-363 cluster) in normal primary T-cell samples as compared to JURKAT and ALL-SIL cell lines transduced with CRISPRi vector and non-targeting (NT) sgRNA or mir-106a-363 targeting sgRNA4.

Supplementary Table 1

miRNA seed family	miRNA cluster	mature miRNA ID	pre-miRNA ID	miRNA sequence	expression level in T-ALL cell lines
miR-17	mir-17-92	hsa-miR-17-5p	hsa-mir-17	C**AAAGUGC**UUACAGUGCAGGUAG	high
	mir-106a-363	hsa-miR-106a-5p	hsa-mir-106a	A**AAAGUGC**UUACAGUGCAGGUAG	high
	mir-106b-25	hsa-miR-106b-5p	hsa-mir-106b	U**AAAGUGC**UGACAGUGCAG - AU -	high
	mir-17-92	hsa-miR-20a-5p	hsa-mir-20a	U**AAAGUGC**UUAUAGUGCAGGUAG	high
	mir-106a-363	hsa-miR-20b-5p	hsa-mir-20b	C**AAAGUGC**U CAUAGUGCAGGUAG	high
	mir-106b-25	hsa-miR-93-5p	hsa-mir-93	C**AAAGUGC**UGUUCGUGCAGGUAG	high
miR-18	mir-17-92	hsa-miR-18a-5p	hsa-mir-18a	U**AAGGUGC**AUCUAGUGCAGAUAG	high
	mir-106a-363	hsa-miR-18b-5p	hsa-mir-18b	U**AAGGUGC**AUCUAGUGCAGUUAG	low
miR-19	mir-17-92	hsa-miR-19a-3p	hsa-mir-19a	U**GUGCAAA**UCUAUGCAAAACUGA	high
	mir-17-92<br>mir-106a-363	hsa-miR-19b-3p	hsa-mir-19b-1<br>hsa-mir-19b-2	U**GUGCAAA**UCCAUGCAAAACUGA	high
miR-92	mir-17-92<br>mir-106a-363	hsa-miR-92a-3p	hsa-mir-92a-1<br>hsa-mir-92a-2	U**AUUGCAC**UUGUCCCGGCCUGU	low
	mir-106a-363	hsa-miR-363-3p	hsa-mir-363	A**AUUGCAC**GGUAUCCAUCUGUA	high
	mir-106b-25	hsa-miR-25-3p	hsa-mir-25	C**AUUGCAC**UUGUCUCGGUCUGA	high
miR-130	-	hsa-miR-130a-3p	hsa-mir-130a	C**AGUGCAA**UGUUAAAAGGGCAU	high
	mir-130b-301b	hsa-miR-130b-3p	hsa-mir-130b	C**AGUGCAA**UGAUGAAAGGGCAU	high
	mir-130b-301b	hsa-mir-301b-3p	hsa-mir-301b	C**AGUGCAA**UGAUAUUGUCAAAGC	low

Mature miRNAs (guide strands) targeted for CRISPRi in our study with indication of miRNAs belonging to common seed families. miRNAs from seed families miR-17, miR-18, miR-19 and miR-92 are encoded within three paralogous miRNA clusters (mir-17-92, mir-106a-363 and mir-106b-25).

The color of nucleotide positions marks differences between miRNAs with high sequence homology.

Bolded and underlined nucleotides form miRNA seed sequences.

The information about expression level of miRNAs in T-ALL cell lines is based on miRNA sequencing [1] and RT-qPCR validation (low expression - lack of amplification in RT-qPCR or poor amplification efficiency).

Reference

1. Wallaert, A.; Van Loocke, W.; Hernandez, L.; Taghon, T.; Speleman, F.; Van Vlierberghe, P. Comprehensive MiRNA Expression Profiling in Human T-Cell Acute Lymphoblastic Leukemia by Small RNA-Sequencing. Sci. Rep. 2017, 7.

Supplementary Table 2. The list of primers and nucleotides used for cloning into expression vectors

Oligonucleotides used for cloning into miRZip miRNA knockdown vector (System Biosciences) for expression of shRNAs

miRZip miR-20b-5p sense	GATCCGCAAAGTACTCATAGTGCAAGTGGCTTCCTGTCAGACTACCTGCACTATGAGCACTTTGTTTTTG
miRZip miR-20b-5p antisense	AATTCAAAAACAAAGTGCTCATAGTGCAGGTAGTCTGACAGGAAGCCACTTGCACTATGAGTACTTTGCG
miRZip miR-130a-3p sense	GATCCGCAGTGTAATGTTAAAAGAGCGTCTTCCTGTCAGAATGCCCTTTTAACATTGCACTGTTTTTG
miRZip miR-130a-3p antisense	AATTCAAAAACAGTGCAATGTTAAAAGGGCATTCTGACAGGAAGACGCTCTTTTAACATTACACTGCG
miRZip miR-130b-3p sense	GATCCGCAGTGTAATGATGAAAGAGCGTCTTCCTGTCAGAATGCCCTTTCATCATTGCACTGTTTTTG
miRZip miR-130b-3p antisense	AATTCAAAAACAGTGCAATGATGAAAGGGCATTCTGACAGGAAGACGCTCTTTCATCATTACACTGCG

EcoRI sticky end

BamHI sticky end

Oligonucleotides used for cloning into pU6-sgRNA-Ef1alpha-Puro-T2A-GFP vector for expression of sgRNAs

mir-17-92 sgRNA 1 sense	TTGCTCCGGTCGTAGTAAAGCGCGTTTAAGAGC
mir-17-92 sgRNA 1 sense	TTAGCTCTTAAACGCGCTTTACTACGACCGGAGCAACAAG
mir-17-92 sgRNA 2 sense	TTGTTCGCGCCACTTCGCGCCCTGTTTAAGAGC
mir-17-92 sgRNA 2 antisense	TTAGCTCTTAAACAGGGCGCGAAGTGGCGCGAACAACAAG
mir-17-92 sgRNA 3 sense	TTGTGGGCGGACGGCGAACACAAGTTTAAGAGC
mir-17-92 sgRNA 3 antisense	TTAGCTCTTAAACTTGTGTTCGCCGTCCGCCCACAACAAG
mir-17-92 sgRNA 4 sense	TTGACGAGGTACCTGCGCGCCAGGTTTAAGAGC
mir-17-92 sgRNA 4 antisense	TTAGCTCTTAAACCTGGCGCGCAGGTACCTCGTCAACAAG
mir-17-92 sgRNA 5 sense	TTGTGCCGCCGGGAAACGGGTTGGTTTAAGAGC
mir-17-92 sgRNA 5 antisense	TTAGCTCTTAAACCAACCCGTTTCCCGGCGGCACAACAAG
mir-17-92 sgRNA 6 sense	TTGTGTGCGACATGTGCTGCCGGGTTTAAGAGC
mir-17-92 sgRNA 6 antisense	TTAGCTCTTAAACCCGGCAGCACATGTCGCACACAACAAG
mir-106a-363 sgRNA 1 sense	TTGCCAACGCGAGTCACTCGCACGTTTAAGAGC
mir-106a-363 sgRNA 1 antisense	TTAGCTCTTAAACGTGCGAGTGACTCGCGTTGGCAACAAG
mir-106a-363 sgRNA 2 sense	TTGAGTATCACGCAGACGTGCCAGTTTAAGAGC
mir-106a-363 sgRNA 2 antisense	TTAGCTCTTAAACTGGCACGTCTGCGTGATACTCAACAAG
mir-106a-363 sgRNA 3 sense	TTGATACAACGCTTCTTAAAGTCGTTTAAGAGC
mir-106a-363 sgRNA 3 antisense	TTAGCTCTTAAACGACTTTAAGAAGCGTTGTATCAACAAG
mir-106a-363 sgRNA 4 sense	TTGTAAGAACAGTACCTTTGAGCGTTTAAGAGC
mir-106a-363 sgRNA 4 antisense	TTAGCTCTTAAACGCTCAAAGGTACTGTTCTTACAACAAG

mir-106a-363 sgRNA 5 sense	TTGATGCAACTTTCGACCTTTAAGTTTAAGAGC
mir-106a-363 sgRNA 5 antisense	TTAGCTCTTAAACTTAAAGGTCGAAAGTTGCATCAACAAG
mir-130a sgRNA 1 sense	TTGGGTATCGAGAGCTTCCCGAAGTTTAAGAGC
mir-130a sgRNA 1 antisense	TTAGCTCTTAAACTTCGGGAAGCTCTCGATACCCAACAAG
mir-130a sgRNA 2 sense	TTGAAGTATTCAAGATGGACGGCGTTTAAGAGC
mir-130a sgRNA 2 antisense	TTAGCTCTTAAACGCCGTCCATCTTGAATACTTCAACAAG
mir-130a sgRNA 3 sense	TTGACCGAGGTTGCGAGGCCGGTGTTTAAGAGC
mir-130a sgRNA 3 antisense	TTAGCTCTTAAACACCGGCCTCGCAACCTCGGTCAACAAG
mir-130b sgRNA 1 sense	TTGACTCGGCGGGTCAGGCGGAGGTTTAAGAGC
mir-130b sgRNA 1 antisense	TTAGCTCTTAAACCTCCGCCTGACCCGCCGAGTCAACAAG
mir-130b sgRNA 2 sense	TTGTCCAGAGTGTATCTAGTGCAGTTTAAGAGC
mir-130b sgRNA 2 antisense	TTAGCTCTTAAACTGCACTAGATACACTCTGGACAACAAG
mir-130b sgRNA 3 sense	TTGCTAGAAGGGGCGCGTCGTCCGTTTAAGAGC
mir-130b sgRNA 3 antisense	TTAGCTCTTAAACGGACGACGCGCCCCTTCTAGCAACAAG
mir-130b sgRNA 4 sense	TTGCGCTAGCCGGCTTTGGACTGGTTTAAGAGC
mir-130b sgRNA 4 antisense	TTAGCTCTTAAACCAGTCCAAAGCCGGCTAGCGCAACAAG
non-targeting control sense	TTGGACGGAGGCTAAGCGTCGCAAGTTTAAGAGC
non-targeting control antisense	TTAGCTCTTAAACTTGCGACGCTTAGCCTCCGTCCAACAAG

BstXI sticky end

BlpI sticky end

Primers for RT-qPCR

*BCL2L11*_Forward	CCACCACTTGATTCTTGCAG
*BCL2L11*_Reverse	GTTGCTTTGCCATTTGGTCT
*PTPRG*_Forward	CACCAGTTCCGTGCTCCATT
*PTPRG*_Reverse	GCCCCAACGTAGCCTTCTG
*ACTB*_Forward	CTTCCTGGGCATGGAGTCC
*ACTB*_Reverse	ATCTTGATCTTCATTGTGCTG
*GAPDH*_Forward	TGGTCACCAAGGGCTGCTT
*GAPDH*_Reverse	AGCTTCCCGTTCTCAGCCTT

Supplementary Table 3. The list of TaqMan Advances miRNA Assays used for evaluation of miRNA expression in RT-qPCR

miRNA ID	Assay ID
hsa-miR-16-5p	477860_mir
hsa-miR-25-3p	477994_mir
hsa-let-7a-5p	478575_mir
hsa-miR-17-5p	478447_mir
hsa-miR-18a-3p	477944_mir
hsa-miR-19a-3p	479228_mir
hsa-miR-20a-5p	478586_mir
hsa-miR-19b-3p	478264_mir
hsa-miR-106a-5p	478225_mir
hsa-miR-20b-5p	477804_mir
hsa-miR-363-3p	478060_mir
hsa-miR-106b-5p	478412_mir
hsa-miR-130a-3p	477851_mir
hsa-miR-130b-3p	477840_mir
